# A Bibliometric Analysis of the Innate Immune DNA Sensing cGAS-STING Pathway from 2013 to 2021

**DOI:** 10.3389/fimmu.2022.916383

**Published:** 2022-06-03

**Authors:** Xuan Shi, Sheng Wang, Yutong Wu, Quanfu Li, Tong Zhang, Keting Min, Di Feng, Meiyun Liu, Juan Wei, Lina Zhu, Wei Mo, Zhuoran Xiao, Hao Yang, Yuanli Chen, Xin Lv

**Affiliations:** Department of Anesthesiology, Shanghai Pulmonary Hospital, School of Medicine, Tongji University, Shanghai, China

**Keywords:** innate immunity, cGAS-STING pathway, bibliometrics, VOSviewer, CiteSpace

## Abstract

**Background and aims:**

Cyclic guanosine monophosphate (GMP)-adenosine monophosphate (AMP) (cGAMP) synthase (cGAS) and stimulator of interferon genes (STING) are key components of the innate immune system. This study aims to evaluate the research of cGAS-STING pathway and predict the hotspots and developing trends in this field using bibliometric analysis.

**Methods:**

We retrieved publications from Science Citation Index Expanded (SCI-expanded) of Web of Science Core Collection (WoSCC) in 1975-2021 on 16 March 2022. We examined the retrieved data by bibliometrix package in R software, VOSviewer and CiteSpace were used for visualizing the trends and hotspots of research on the cGAS-STING pathway.

**Results:**

We identified 1047 original articles and reviews on the cGAS-STING pathway published between 1975 and 2021. Before 2016, the publication trend was increasing steadily, but there was a significant increase after 2016. The United States of America (USA) produced the highest number of papers (Np) and took the highest number of citations (Nc), followed by China and Germany. The University of Texas System and Frontiers in Immunology were the most prolific affiliation and journal respectively. In addition, collaboration network analysis showed that there were tight collaborations among the USA, China and some European countries, so the top 10 affiliations were all from these countries and regions. The paper published by Sun LJ in 2013 reached the highest local citation score (LCS). Keywords co-occurrence and co-citation cluster analysis revealed that inflammation, senescence, and tumor were popular terms related to the cGAS-STING pathway recently. Keywords burst detection suggested that STING-dependent innate immunity and NF-κB-dependent broad antiviral response were newly-emerged hotspots in this area.

**Conclusions:**

This bibliometric analysis shows that publications related to the cGAS-STING pathway tend to increase continuously. The research focus has shifted from the mechanism how cGAS senses dsDNA and cGAMP binds to STING to the roles of the cGAS-STING pathway in different pathological state.

## Introduction

Over the past two decades, in mammalian cells, recognition of pathogens’ nucleic acids has been a key feature to sense microbial pathogens. In the field of sensing double-stranded DNA (dsDNA), cGAS is an important DNA-binding protein that represents the initiator of sensing dsDNA. Three strategies have been reported for cGAS to recognize pathogens efficiently. Firstly, cGAS is discovered in the cytoplasm, plasma membrane, and nucleus, it can rapidly recognize DNA and initiate the downstream immune response (1–4). Secondly, the recognition would be strengthened by high-mobility group box 1 protein (HMGB1), mitochondrial transcription factor A (TFAM) and modified by reactive oxygen species (ROS) (5, 6). Thirdly, the second messenger cyclic GMP-AMP (cGAMP) from these infected cells would show alarm to bystander cells to activate cGAS-STING pathway in these cells (7). When combined with dsDNA, the structure of cGAS would change and affect catalytic pockets. ATP and GTP in this pocket are catalyzed by cGAMP (8). As a second messenger, cGAMP is detected by STING, a cyclic-dinucleotide sensor (9, 10). Then STING is transported from the endoplasmic reticulum (ER) to Golgi through ER-Golgi intermediate compartment and sets off downstream signaling reaction (10, 11). STING is regarded as the central molecule of the downstream of I IFN (12, 13). STING is reported to enhance the activity of RIG-1-like receptors (RLR) signaling pathway (14) and the activity of interferons-β (IFN-β) which is dependent on interferon regulator factor 3 (IRF3) (15–17). In addition, the activation of STING can activate TANK-binding kinase 1 (TBK1) and IκB kinase (IKK). p-TBK1 phosphorylates interferon regulatory factor 3 (IRF3), and IRF3 translocated to the nucleus to transcript IFN-I (18). IKK is also recruited by STING, phosphorylates IκBα and induces NF-κB to translocate to nucleus. After that, lots of cytokines are transcribed to induce inflammatory and immune responses (19). In recent years, scholars have done lots of research about the cGAS-STING pathway. It is important to explore the hotspots and development trends of the cGAS-STING pathway in the past 10 years with CiteSpace and VOSviewer software.

Bibliometric analysis is a useful method by which scholars can evaluate the history, current, and future of publications and their quantity and quality (20). Bibliometrics can analyze publications (books, journals, and so on) by applying the literature system and metrology as objects. In addition, it can provide useful information to help to write the guideline, make decisions and treat diseases (21–23). In these years, many bibliometric analyses have been published in the biological field. However, bibliometric analysis on the cGAS-STING pathway remains a void. So the study aims to systematically analyze the research on the cGAS-STING pathway to digest the current state and the hotspots in this field.

## Materials and Methods

### Data Collection

**Figure 1:**
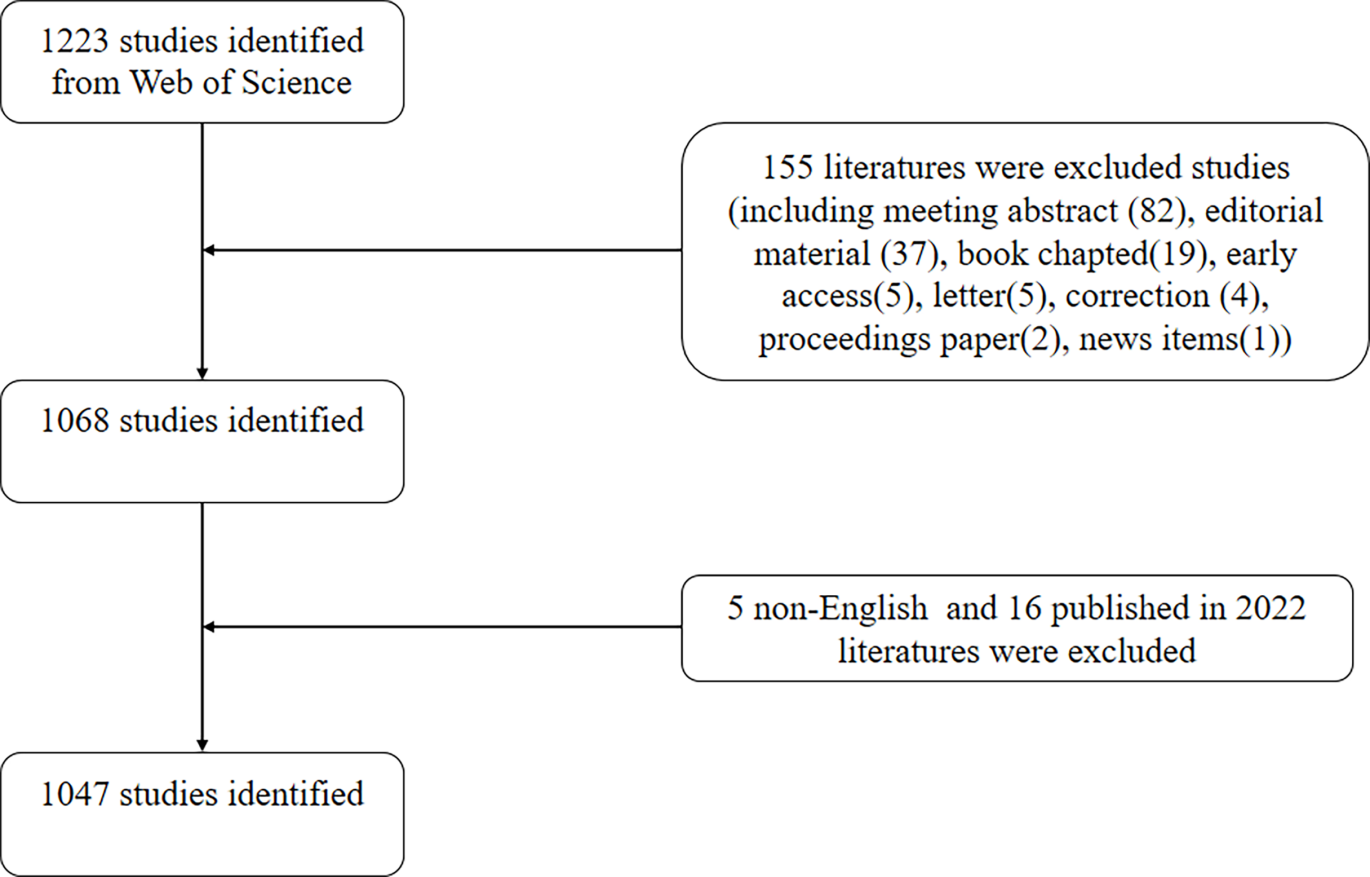
Flowchart of the screening process.

The Science Citation Index Expanded (SCI-expanded) of Web of Science Core Collection (WoSCC) in 1975-2021 was systematically searched from 1 January 1975 to 31 December 2021 and was downloaded in a single day (2022.03.17) to avoid deviations. The search terms were set as follows: TS = (“stimulator of interferon genes” OR “transmembrane protein 173” OR STING OR ERIS OR MITA OR MPYS OR NET23 OR TMEM173) AND TS = (“Mab-21 domain containing 1” OR “E330016A19Rik” OR “cyclic guanosine monophosphate-adenosine monophosphate synthase” OR “cGAMP synthase” OR “cyclic GMP-AMP synthase” OR “MB21D1” OR “cGAS”). Two reviewers (XS and YW) independently identified these data search and then discussed the potential differences, the final agreement reached 0.90 (24). These two reviewers then sent these original articles and reviews into Endnote for further validation. Finally, 1047 original articles and reviews written in English were included. **Figure 1** is the flowchart of literature selection.

Duplicate authors and misspelled elements were removed, and we used a thesaurus file to merge duplicates into one word, delete the useless words and correct the misspelled elements. Then, the clean data were imported to VOSviewer v.1.6.15.0, CiteSpace version 5.8.R3, and the “bibliometrix package 3.2.1” of R software (Version 4.1.3) for bibliometric analysis.

### Bibliometric Analysis

We used the numbers of papers and citations to represent the bibliographic material as previously reported (25). The productivities of papers were represented by the numbers of publications (Np), the impacts were represented by the numbers of citations (without self-citations) (Nc) and the numbers of average citations (Na) were Nc/Np, which represented the qualities of publications. These elements were regarded as three main perspectives to evaluate the levels of researches. In some cases, H-index was also developed to evaluate individual academic achievements, the publication output of a region or a nation, an institution, or a journal (26). What’s more, the impact factor (IF) from the latest version of Journal Citation Reports (JCR), and local citation score (LCS) also indicated the value of an article (27, 28).

VOSviewer, CiteSpace, and R (Version 4.1.3) are used for statistical computing and graphics. VOSviewer is a program to establish bibliometric maps by using the data collected from Web of Science Core Collection (29). It can provide a general comprehensive and detailed view of bibliometric maps based on collaborative data. CiteSpace is a program to analyze the potential knowledge contained in the scientific literature and visualize collected data (30). R software (Version 4.1.3) is the language and environment, which is wildly used for statistical computing and graphics (31). In this study, the bibliometrix package 3.2.1 in R was used to analyze data and perform a basic bibliometric analysis (32).

## Results

### An Overview of Publications on the cGAS-STING Pathway

The number of original articles (785) and reviews (262) published was 1047, the total Nc for retrieved articles and reviews was 33357, the average Nc per article was 31.86. The H-index of all original articles and reviews was 102.

### The Annual Trend of Paper Publication Quantity

**Figure 2:**
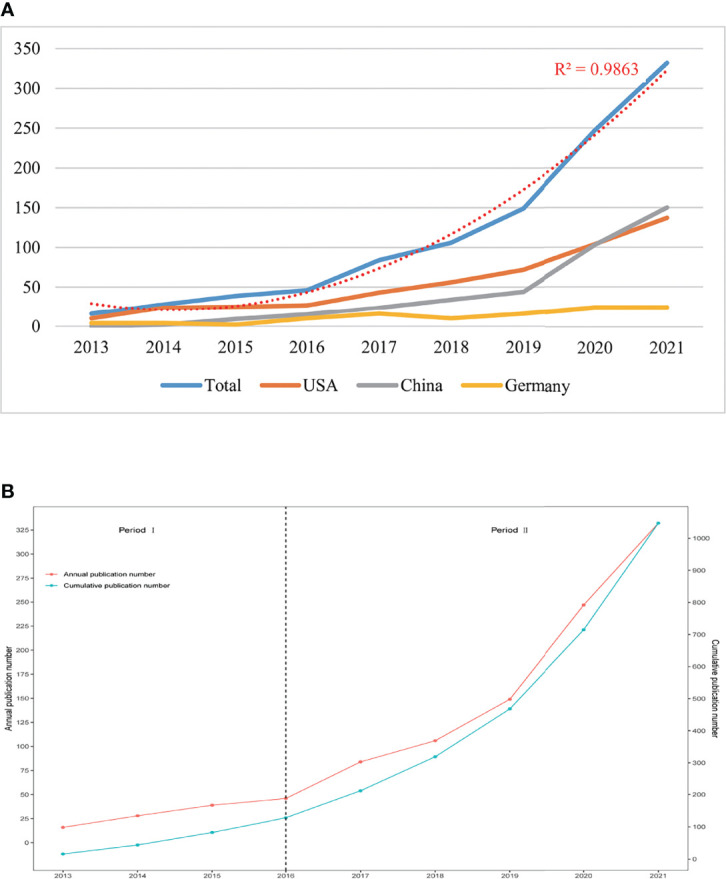
**(A)** The total numbers of publications and top three countries from 2013 to 2021. **(B)** The numbers of publications by year and accumulation from 2013 to 2021.

The annual Np related to the cGAS-STING pathway was shown in **Figure 2A**. The numbers of annual papers rose rapidly from 16 in 2013 to 332 in 2021 and the correlation coefficient R^2^ is 0.9863. The rapid increase indicated that more and more researchers were paying attention to this area.

From 2013 to 2021, the Np in the USA had increased steadily. When it comes to China, before 2019, the Np was at a low level and was almost as half as that in the USA. However, the Np in China had reached the first place in 2021. This might be related to the increased investments of the Chinese government in scientific research.

In **Figure 2B**, it was interesting to note that the number of annual publications can be divided into two stages. With the model of research development (33), we found that from 2013 to 2016 (period I), publications outputs were at a low level. Theories in this area were not completed and the cGAS-STING pathway just began to come into focus. From 2016 to now (period II), a rapid increase occurred and the publication outputs had been over 1000 in 2021, which represented that more scholars were conducting research in this field and theories about the cGAS-STING pathway were booming. Since the cGAS-STING pathway has attracted more attention, a spurt would occur shortly.

### Analysis of Countries and Affiliations

**Figure 3:**
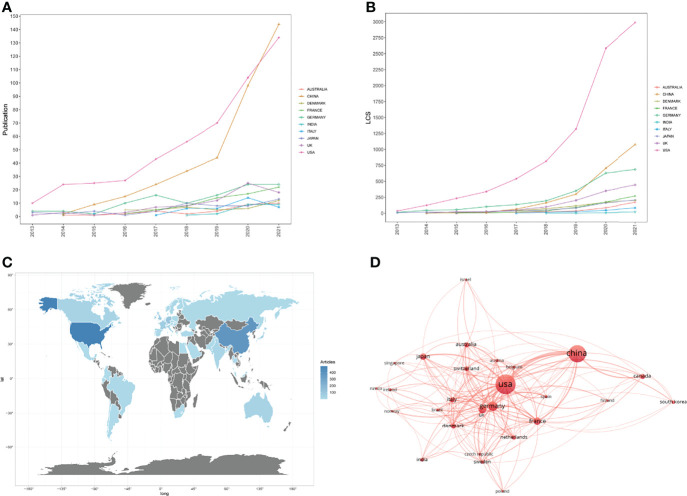
**(A)** Publications in the 10 most productive countries/regions. **(B)** The yearly number of local citation scores (LCS) of the 10 most productive countries/regions. **(C)** Distributions of countries/regions of publications. **(D)** The cooperation map of countries involved in the cGAS-STING pathway.

A total of 1047 articles were published from 54 countries and regions. We ranked the top 10 output countries and regions of all authors according to the number of Np (**Table 1**). Because we used the bibliometrix package in R (Version 4.1.3) software to analyze all data, the data of England, Scotland, and Wales were merged automatically by the package in analysis of countries, and finally these were shown as UK. In **Figure 3A**, the Np in other countries was relatively at low levels and remained steady except the USA and China. China ranked first in Np in 2021 but the LCS of China was much lower than that of the USA (**Figure 3B**), representing that qualities of publications in China were still at a relative low level. The Np and LCS in the USA both increased rapidly, which means the publications about the cGAS-STING pathway in the USA were not only for quantity but also for quality. **Figure 3C** represents the distributions of publications in different countries and regions. Cooperation among different countries is an important driving force to promote the development of scientific research. To this point, close cooperation among different countries were shown in **Figure 3D**. The lines donated the cooperation between countries. The wider the lines, the closer the cooperation. However, most countries lacked lines, which means they lacked stable cooperation and communication.

**Table 1:** Publications in the 10 most productive countries/regions.

Rank	Country/Region	(Np)	% of (1047)	(Nc)	(Na)	H-index
1	USA	493	47.09	28654	58.12	88
2	China	370	35.34	7574	20.47	48
3	Germany	110	10.51	6680	60.73	40
4	UK	82	7.83	5011	61.11	33
5	France	69	6.59	2845	41.23	25
6	Japan	53	5.06	2453	46.28	25
7	Denmark	42	4.01	2007	47.79	19
8	Italy	36	3.44	912	25.33	15
9	Australia	33	3.15	1846	55.94	17
10	Canada	33	3.15	1379	41.79	13

**Table 2** showed the top 10 affiliations with the highest number of publications related to the cGAS-STING pathway. University of Texas System had the highest Np (88, 8.40%) among all affiliations, which were almost as twice as Chinese Academy of Sciences (51, 4.87%). The team of Zhijian James Chen, who discovered cGAS for the first time and explained its function, is from University of Texas System. The publications from this team contributed a lot for the first place of University of Texas System. The Nps of affiliations ranking three to five were the same, Howard Hughes Medical Institute (48, 4.58%), University of California System (48, 4.58%), and Harvard University (48, 4.58%). Among the top 10 affiliations, half of them belonged to the USA. This was related to its high investment and strong technical strength. In addition, University of Texas System got the highest H-index (38) followed by Howard Hughes Medical Institute (37), scholars in this area should focus on their high-quality research notably.

**Table 2:** The top 10 productive affiliations.

Rank	Affiliations	Country	(Np)	(Nc)	(Na)	H-index
1	UNIV OF TEXAS SYSTEM	USA	88	10905	123.92	38
2	CHINESE ACADEMY OF SCIENCES	China	51	1120	21.96	21
3	HOWARD HUGHES MEDICAL INSTITUTE	USA	48	12049	251.02	37
4	UNIV OF CALIFORNIA SYSTEM	USA	48	3151	65.65	24
5	HARVARD UNIV	USA	48	836	17.42	22
6	WUHAN UNIVERSITY	China	36	671	18.64	15
7	CENTRE NATIONAL DE LA RECHERCHE SCIENTIFIQUE CNRS	France	35	1130	32.29	18
8	INSTITUTION NATIONAL DE LA SANTE ET DE LA RECHERCHE MEDICALE INSERM	France	34	1900	55.88	17
9	AARHUS UNIVERSITY	Denmark	33	1715	51.97	18
10	NATIONAL INSTITUTES OF HEALTH NIH USA	USA	28	1591	56.82	14

### Analysis of Journals

The 10 journals with the most research in cGAS-STING area, along with H-index and impact factor (IF) Eigenfactor Score as indicators of impact were listed in **Table 3**. These journals were more likely to accept articles on cGAS-STING pathway because they had produced the most publications on the related topics recently. Scholars in cGAS-STING area should focus on research published in these journals. The highest IF belonged to Nature (IF=49.926), followed by Immunity (IF=31.745), Cell Host & Microbe (IF=21.023), Nature Communication (IF=14.919) and Proceedings of the National Academy of Sciences of the United States of America (PNAS) (IF=11.205). The IF of these 5 journals were over 10 and they published over 1/10 papers in this area in the past, representing that cGAS-STING is a popular research orientation and it is not difficult for studies in cGAS-STING area to publish in top journals.

**Table 3:** The top 10 productive journals.

Rank	Journal	Np	H-index	Nc	Na	IF (2020)
1	FRONTIERS IN IMMUNOLOGY	55	10	338	6.15	7.561
2	JOURNAL OF IMMUNOLOGY	35	17	892	25.49	5.442
3	JOURNAL OF VIROLOGY	31	18	915	29.52	5.078
4	PLOS PATHOGENS	31	17	826	26.65	6.823
5	NATURE COMMUNICATIONS	29	18	2149	74.10	14.919
6	CELL REPORTS	27	18	1838	68.07	9.423
7	NATURE	27	24	6393	236.78	49.962
8	PNAS	25	17	1322	52.88	11.205
9	IMMUNITY	17	15	3159	185.82	31.745
10	CELL HOST MICROBE	13	13	1514	116.46	21.023

### Analysis of Local Citation Score

**Figure 4:**
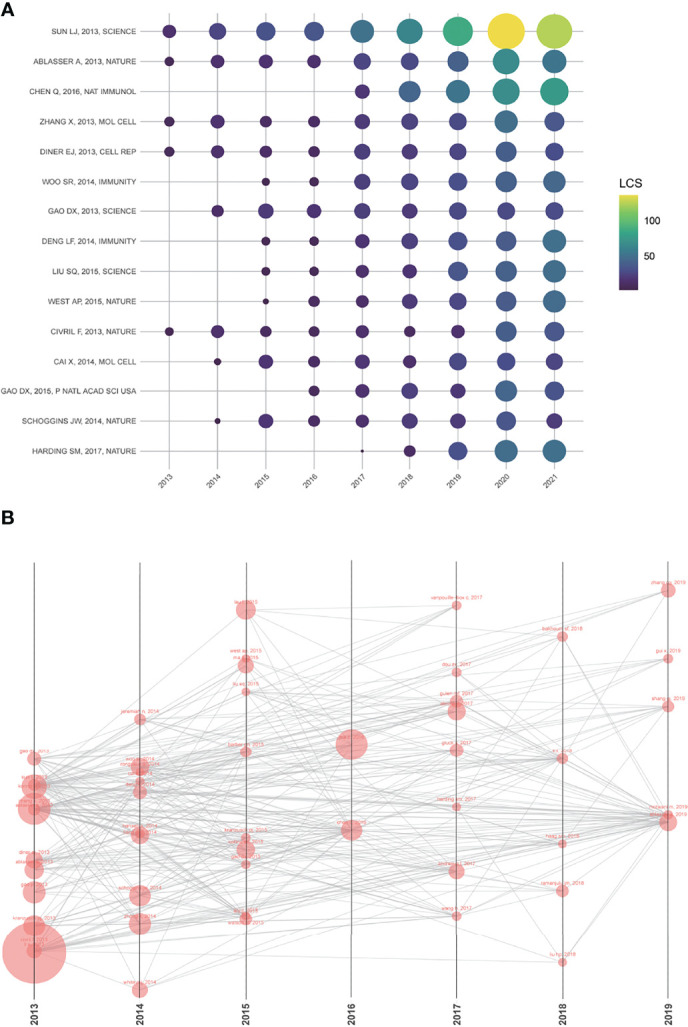
**(A)** The yearly number of local citations of papers with high local citation scores (LCS). The size and colors of the circle present the LCS of papers in that year. **(B)** One paper cited by the other papers with the top 50 LCS. The size of each node is proportional to the frequency of this article by the other 49 articles.

The LCS analysis provided detailed information for articles with high local citations. The numbers of LCS per year for the top 15 articles were presented in **Figure 4A** and **S Table 1**. Interestingly, 8 of them were from the team of Zhijian James Chen, the pioneer and founder of the cGAS-STING area. These research outputs of Chen’s lab were leading the trend and breakthrough in this area. The paper written by Sun LJ, the Ph.D. student in Chen’s lab, got the highest LCS score (555). In this paper, the authors firstly discovered an enzyme named cyclic GMP–AMP synthase (cGAS), which can detect DNA and active I IFN signaling pathway (1). Apart from two reviews (Chen Q, 2016; Harding SM, 2017) (8, 34), the other 13 high LCS studies were all from 2013 to 2015, when the area just emerged. After 2016, scholars around the world are conducted more and more research based on these classical research. Visualization of the top 50 LCS articles was shown in **Figure 4B**. In this network, each node represented a cited article, the size of each node was proportional to the frequency of this article by the other 49 articles.

### Analysis of Hotspots and Frontiers

**Figure 5:**
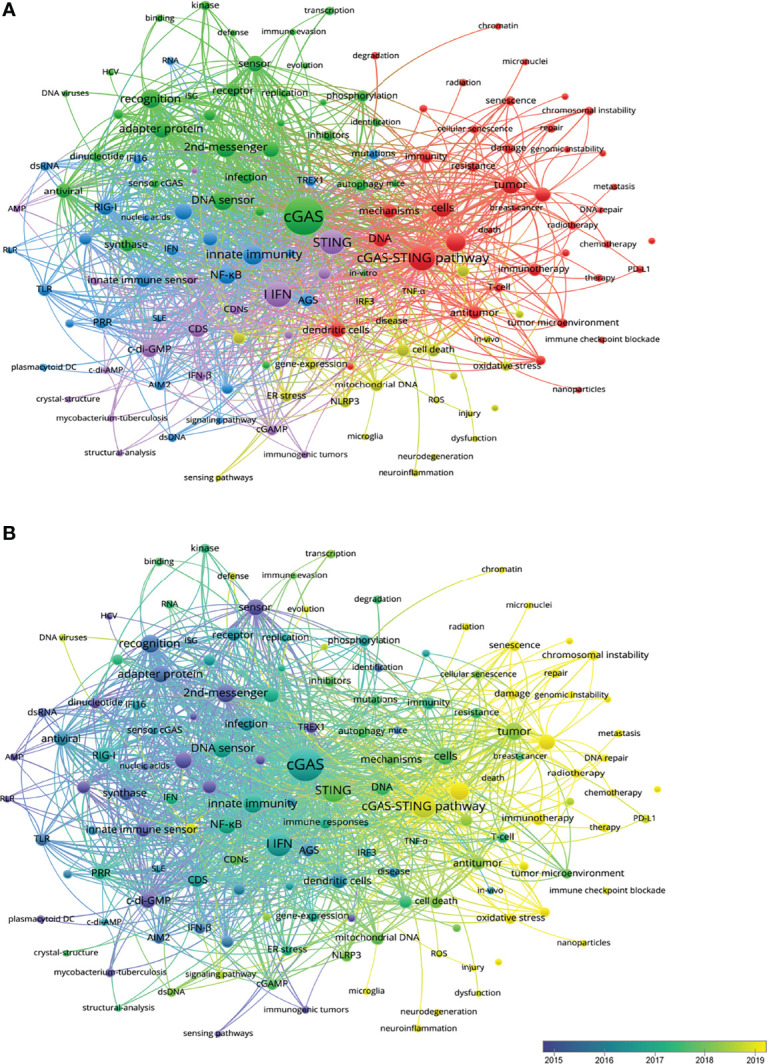
**(A)** CiteSpace visualization map of keywords clustering analysis related to the cGAS-STING pathway. The size of each nodes represents the frequency of occurrences. **(B)** Visualization of keywords according to the average publication year. Keywords in yellow appeared later than that in blue.

By analyzing keywords, readers can easily summarize the topic of one study and explore the hotpots and directions in this area. Thus, co-occurrence analysis described of hot topics in this area (**Figure 5A**). There were five clusters: cGAS-STING pathway in inflammation and tumor immunology (red), cGAS-STING pathway sensing virus and its structure foundation (green), cGAS-STING pathway in innate immunity (purple), in ROS-induced inflammasome activation (yellow), and in autoimmune disease (blue). In overlay visualization, keywords were colored differently according to their average publication year (**Figure 5B**). For instance, ‘DNA sensor’ and ‘2nd-messenger’ were appeared at the beginning of the discovery of this area, whereas keywords ‘Inflammation’ and ‘Tumor’ were more recent. Senescence (cluster1, APY: 2020.143), Cancer therapy (cluster2, 2020), Neuroinflammation (cluster3, APY: 2020.539), and Neurodegeneration (cluster3, APY: 2020.3) are colored in yellow, which indicated that these fields had grown in popularity recently and would become hotspots soon (**S Table 2**).

### Co-cited Reference Clusters Analysis

**Figure 6:**
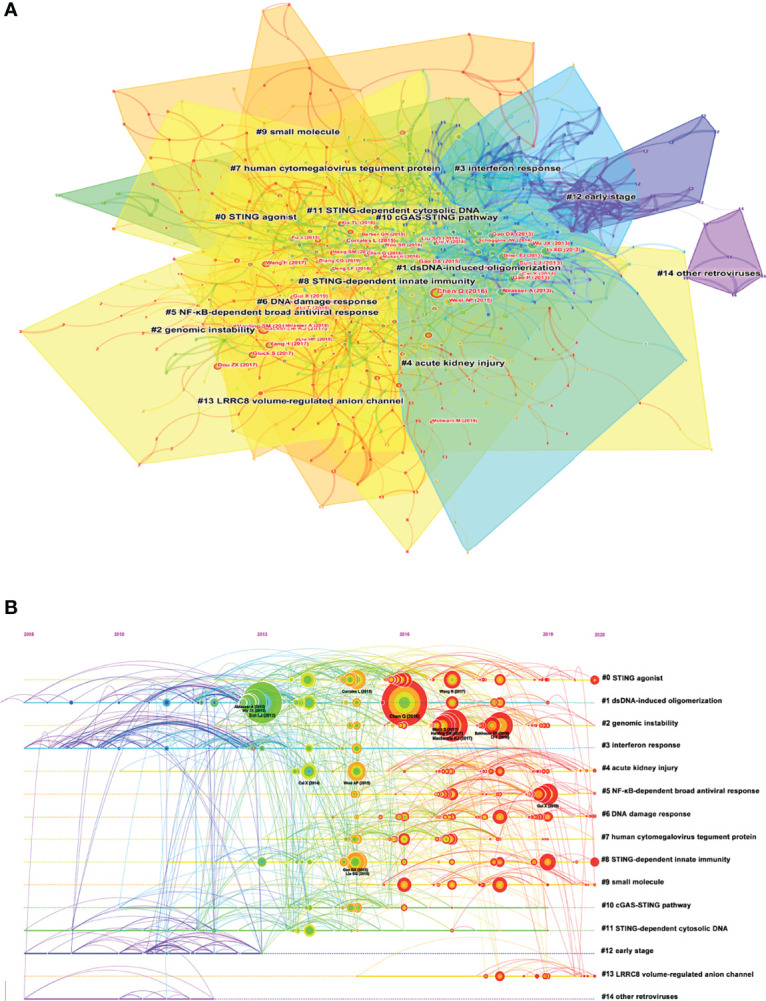
**(A)** The clustered network map of co-cited references on the cGAS-STING pathway. **(B)** The timeline view of co-citation clusters with their cluster-labels on the right.

A co-citation network is a network of references co-cited by one or more papers at the same time. Conceptual clusters were created when a set of manuscripts were cited repeatedly together. **Figure 6A** showed the different clusters of these co-cited references, and 15 clusters were divided by CiteSpace: STING agonist, dsDNA-induced oligomerization, genomic instability, interferon response, acute kidney injury, NF-κB-dependent broad antiviral response, DNA damage response, human cytomegalovirus tegument protein, STING-dependent innate immunity, small molecule, cGAS-STING pathway, STING-dependent cytosolic DNA, early stage, LRRC8 volume-regulated anion channel, and other retroviruses. A timeline view of distinct clusters was presented in **Figure 6B**. It showed that cluster 1, dsDNA-induced oligomerization, had the most citation burst. Moreover, the hotspots were shifting from dsDNA-induced oligomerization to genomic instability, STING-dependent innate immunity, and NF-κB-dependent broad antiviral response.

### Burst Detection

**Figure 7:**
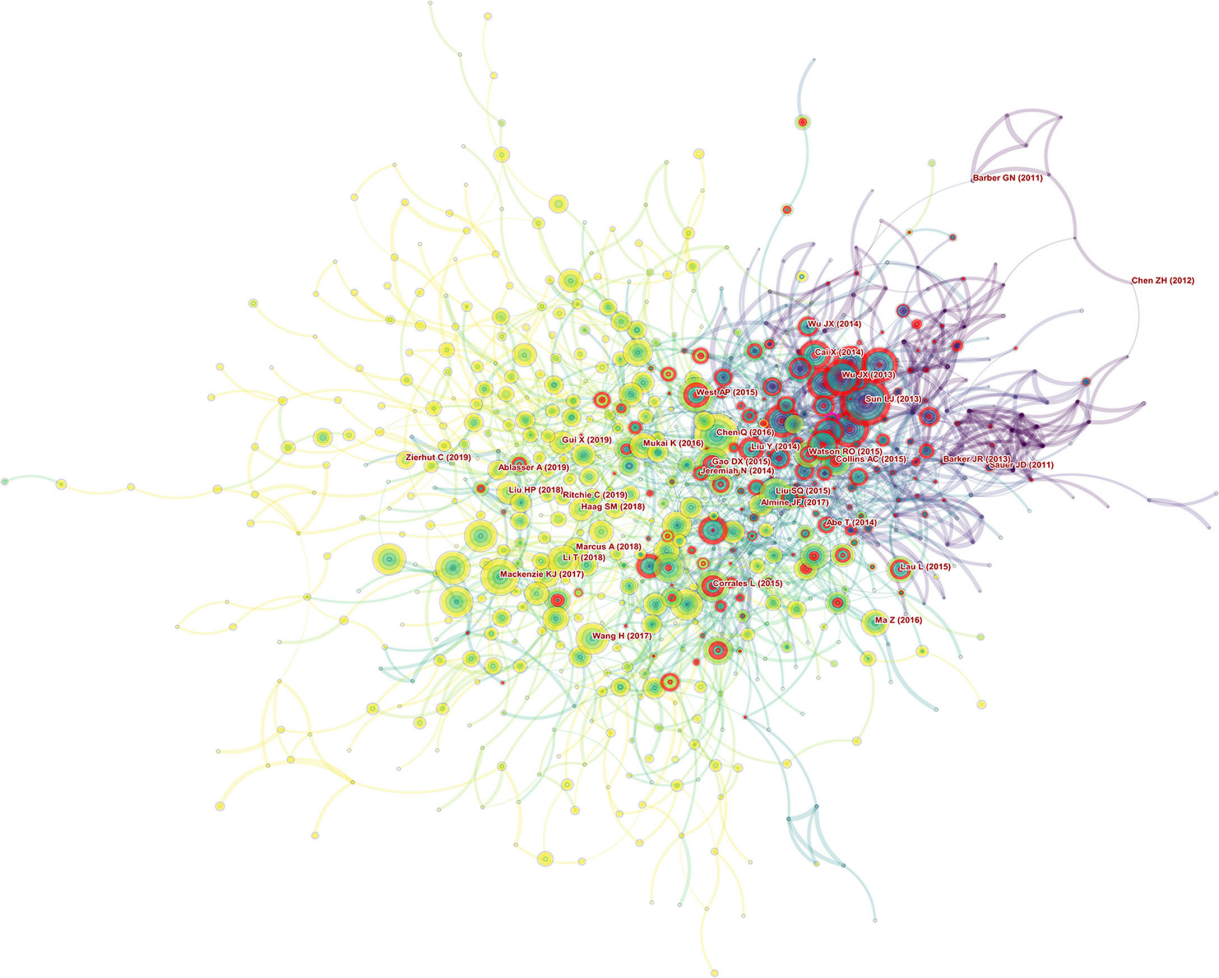
Papers with the strongest citation bursts in original articles on the cGAS-STING pathway between 2013 and 2021.

**Figure 8:**
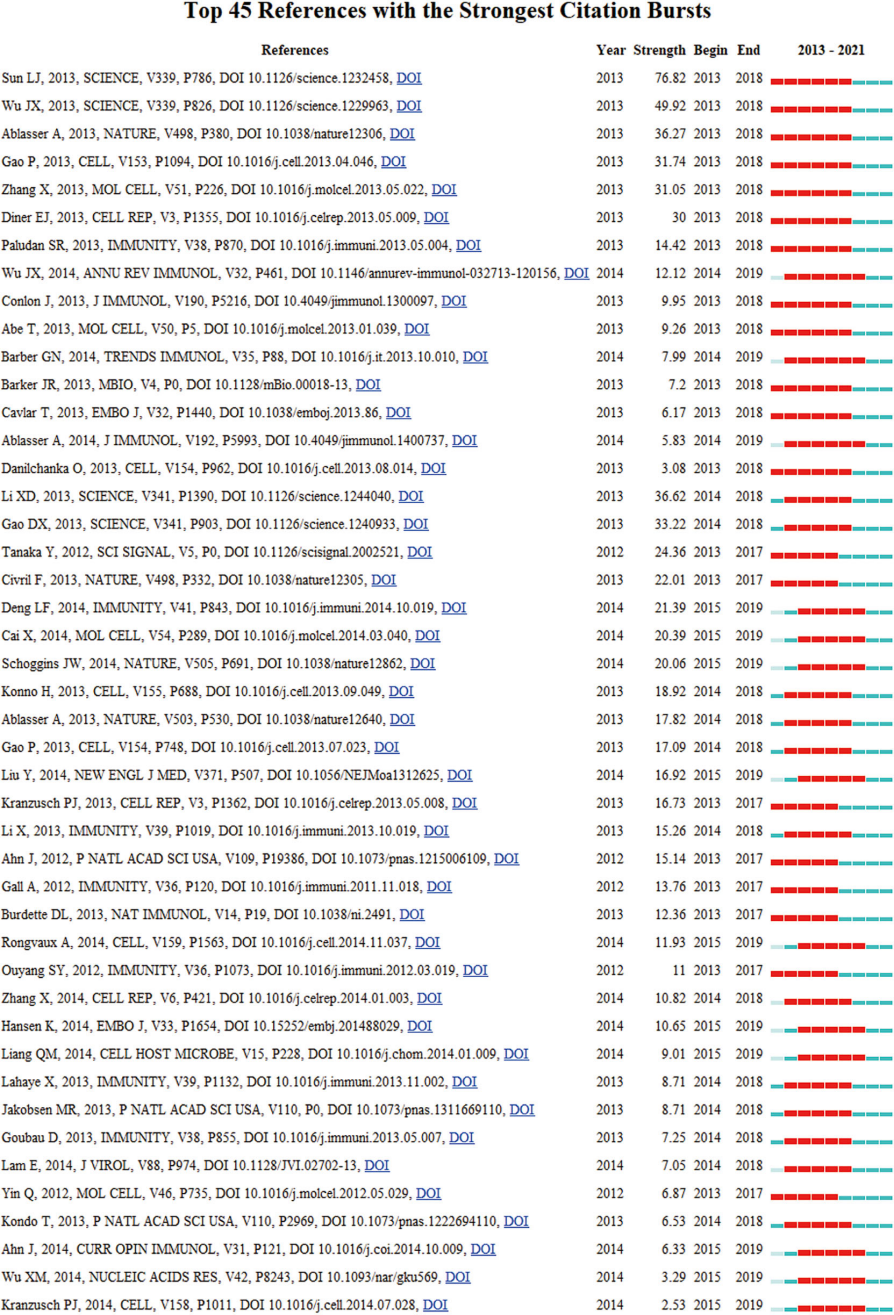
CiteSpace visualization map of the top 45 references with the strongest citation bursts involved in the cGAS-STING pathway.

Burst detection is used to reveal the hot references with an abrupt increase over time. In **Figure 7**, nodes represented articles, those nodes with red circles represented burst articles in this area. **Figure 8** showed the most burst of co-cited references began in 2013, the year when the team of Zhijian James Chen discovered cGAS-STING pathway. 4 of 5 top strongest citation bursts were from the team of Zhijian James Chen, which also indicated his team’s great influence in this field.

## Discussion

In this study, we analyzed the development trends and hotspots of research on the cGAS-STING pathway by VOSviewer, CiteSpace and R (Version 4.1.3) software. We retrieved 1047 original articles and reviews published in 2013-2021. The annual numbers of publications showed an overall upward tread. Interestingly, the Np rocketed up after 2016. These publications with high LCS published before 2016 were the main reason for the rapid growth of the annual Np after 2016.

Among the top countries/regions, the USA ranked first in Np (493, 47.09%), followed by China (370, 35.34%), indicating that the USA and China are the leading countries in the cGAS-STING area. This was closely related to the large research expenditures of the USA and China in recent years. However, the Np, Na, and H-index in the USA were all higher than those in China. This may be because the cGAS-STING pathway was initially proposed by Zhijian James Chen (1), and then deep and extensive research were conducted by Chen’s team in the cGAS-STING area. What’s more, five of the top ten affiliations and seven of the top ten journals were from the USA. Because of these, the USA prevails in the cGAS-STING area. When it comes to affiliations, the H-index of Chinese Academy of Sciences (21) and Wuhan University (15) were similar to the other top 10 affiliations except for University of Texas System (38), but only one original study was included in the top 50 LCS (35), symbolizing that there were good studies in China which attracted the attention of international counterparts, but scholars and affiliations in China should make more efforts to promote the quality of their studies in this field. The other countries also made contributions to this field, although their influences of them were not as good as those in the USA and China.

As can be seen in the co-occurrence network, there were many lines from the USA and the line between the USA and China was the thickest, indicating that the collaboration between the USA and China was very close. Moreover, there were close collaborations between the USA and some countries in Europe, with 3 European affiliations listed in the top 10 affiliations. Therefore, institutions in the USA, China, and other countries should remove academic barriers, try to communicate to promote the development of the cGAS-STING pathway.

Notably, of the top 10 productive journals, the IFs of five were over 10, and the number of published papers in these journals accounted for 1/10 of that in the cGAS-STING area. This indicated that studies about the cGAS-STING pathway were of high quality. Frontiers in immunology (55, 5.33%) published the most articles in this area, followed by journal of immunology (35, 3.39%) and journal of virology (30, 2.91%), which reminded scholars to pay more attention to the roles of the cGAS-STING pathway in immunity and virus detection. In addition, the burst detection showed that STING-dependent innate immunity and NF-κB-dependent broad antiviral response were the hotspots recently. Scholars on this topic should pay more attention to these hotspots.

In the initial phase of one field, research is focused on the basic theories and mechanisms, which lay a solid foundation for further studies. Similarly, the hotspots of the cGAS-STING pathway have been changed from the mechanism to its roles in different diseases and translational medicine. In the first few years, scholars such as Zhijian James Chen and Veit Hornung discovered the role of cGAS in sensing dsDNA and activating the I IFN pathway. cGAS activates the second-messenger (36), which is essential for the STING activation (37–39). What’s more, scholars analyzed the structural mechanism how cGAS senses cytosolic DNA (40, 41).

In the next stage, scholars started to study the roles of the cGAS-STING pathway in different diseases and the influences of the cGAS-STING pathway in cell life activities. Immunity published two studies to demonstrate the roles of the cGAS-STING pathway in immunogenic tumors (42, 43), initiating studies of the cGAS-STING pathway in diseases. In these studies, the STING pathway was regarded as a key regulator of tumor immune responses. Researchers found that tumor-derived DNA was the ligand of STING pathway and was associated with phosphorylation of TBK1 and IRF3 and STING-dependent IFN-β. In STING-deficient mice, most of the therapeutic effects for the immune inhibitory factors were lost. In 2015, the relationships between the cGAS-STING pathway and apoptosis, autophagy, and inflammasome activation were studied by scholars (44–46). Based on these mechanistic investigations, the team of Zhijian James Chen and Blossom Damania reviewed the roles of the cGAS-STING pathway in autoimmune, inflammatory disease, and virus infection, respectively (8, 47). These two reviews concluded the studies between 2013 and 2016 and thus got high LCS.

In recent years, the keywords have focused on the roles of the cGAS-STING pathway in the treatments of diseases. In this period (period II in **Figure 2B**), publications increased rapidly based on previous studies. Article keywords demonstrated that scholars in the fields of cancer and neuroscience should pay more attention to the cGAS-STING pathway because these were hotspots in recent years. Shannon Grabosch’s study demonstrated that cisplatin activated the cGAS-STING pathway to modify tumor immunogenicity by increasing PD-L1, MHC I and calreticulin in tumor cells (48). In malignant tumors, scientists found that the expression of STING was positively correlated with immune cell infiltration (49). Inhibition of cGAS and STING in tomor cells can prevent tumor metastasis (50, 51). Scientists also found that cGAS-STING pathway promoted tumor progression in lewis lung cancer (LCC) (52), brain tumor (50), colon tumor (53), oral cancer (54), and tongue squamous cell carcinoma (55). In December 2017, Chukwuemika Aroh et al. firstly demonstrated that administration of cGAMP delivered by ultra-pH-sensitive nanoparticle can induce potent antiretroviral response against HIV-1 isolates (56). After that, more and more researchers paid attention to the nanoscience. Since the nanoparticle is a hotspot recently, with the development of interdisciplinary research, researchers should focus on the effects of the nanoparticle on diseases by interfering with the cGAS-STING pathway.

Based on VOSviewer, CiteSpace and R (Version 4.1.3) software, we analyzed and made the visualization of the literature, and revealed the development trends and the hotspots in this field. At the same time, we used LCS to find the important literature, which led to the development of the cGAS-STING area and scholars in this field should pay close attention to these literature. Moreover, this study provided a better insight into the evolving research foci and trends when compared with traditional reviews. However, there are still some limitations. Firstly, only English articles and reviews from SCI-expanded were included. Secondly, because VOSviewer could not analyze the full texts of the publications, it may omit some information. Lastly, the publications included were from 2013 to 2021, the influential studies published in 2022 with low Nc were excluded, but this limitation would not change the results in this study. Therefore, future work should expand the research base to include non-English studies and the latest outstanding publications.

## Conclusion

This bibliometric analysis revealed that the research on the innate immune DNA sensing cGAS-STING pathway were developing rapidly at present. The USA and China were the leading countries, and the USA has made many outstanding breakthroughs in this field. About 10% studies were published in high-quality journals. From 2013 to 2021, the foci of research on the cGAS-STING pathway has changed from the basic mechanism to treatments of diseases *via* the cGAS-STING pathway, especially cancer and nanoparticle, these would be hotspots of research recently and in the near future.

## Data Availability Statement

The raw data supporting the conclusions of this article will be made available by the authors, without undue reservation.

## Author Contributions

XS and SW conceived the study. XS, YW, QL, TZ, KM, DF, ML, and JW were involved in the data collection and analysis. LZ, WM, ZX, and HY re-examined the data. XS and SW drafted the manuscript. YC and XL revised the manuscript. All authors have provided final approval of the version to be submitted. XS, SW, and YW contributed equally to this work.

## Funding

This work was supported by grants from the National Natural Science Foundation of China (No.81871601, No.82100090, and No.82000085), grants from the Program of Shanghai Academic Research Leader (No.21XD1402800), grants from the Shanghai “Rising Stars of Medical Talent” Youth Development Program: Outstanding Youth Medical Talents, grants from the Shanghai Natural Science Foundation (22ZR1452200), grants from the Shanghai Sailing Program (21YF1438400).

## Conflict of Interest

The authors declare that the research was conducted in the absence of any commercial or financial relationships that could be construed as a potential conflict of interest.

The reviewer, DY, declared a shared affiliation with the authors to the handling editor at the time of review.

## Publisher’s Note

All claims expressed in this article are solely those of the authors and do not necessarily represent those of their affiliated organizations, or those of the publisher, the editors and the reviewers. Any product that may be evaluated in this article, or claim that may be made by its manufacturer, is not guaranteed or endorsed by the publisher.
