# Supplementary material for: A Bibliometric Analysis of the Innate Immune DNA Sensing cGAS-STING Pathway from 2013 to 2021
Source: Front Immunol. 2022 Jun 3;13:916383. doi: 10.3389/fimmu.2022.916383 (PMC9204223; doi:10.3389/fimmu.2022.916383)
Supplement: Supplementary file 1 [file Table_1.docx]

**S Table 1.** The top 15 highly cited publications in Figure 4A.

| Rank | Title | Journal | Year | First Author |
| --- | --- | --- | --- | --- |
| 1 | Cyclic GMP-AMP synthase is a cytosolic DNA sensor that activates the type I interferon pathway | SCIENCE | 2013 | Lijun Sun |
| 2 | cGAS produces a 2'-5'-linked cyclic dinucleotide second messenger that activates STING | NATURE | 2013 | Andrea Ablasser |
| 3 | Regulation and function of the cGAS-STING pathway of cytosolic DNA sensing | NATURE IMMUNOLOGY | 2016 | Qi Chen |
| 4 | Cyclic GMP-AMP containing mixed phosphodiester linkages is an endogenous high-affinity ligand for STING | MOLECULAR CELL | 2013 | Xu Zhang |
| 5 | The innate immune DNA sensor cGAS produces a noncanonical cyclic dinucleotide that activates human STING | CELL REPORTS | 2013 | Elie J Diner |
| 6 | STING-dependent cytosolic DNA sensing mediates innate immune recognition of immunogenic tumors | IMMUNITY | 2014 | Seng-Ryong Woo |
| 7 | Cyclic GMP-AMP synthase is an innate immune sensor of HIV and other retroviruses | SCIENCE | 2013 | Daxing Gao |
| 8 | STING-Dependent Cytosolic DNA Sensing Promotes Radiation-Induced Type I Interferon-Dependent Antitumor Immunity in Immunogenic Tumors | IMMUNITY | 2014 | Liufu Deng |
| 9 | Phosphorylation of innate immune adaptor proteins MAVS, STING, and TRIF induces IRF3 activation | SCIENCE | 2015 | Siqi Liu |
| 10 | Mitochondrial DNA stress primes the antiviral innate immune response | NATURE | 2015 | A Phillip West |
| 11 | Structural mechanism of cytosolic DNA sensing by cGAS | NATURE | 2013 | Filiz Civril |
| 12 | The cGAS-cGAMP-STING pathway of cytosolic DNA sensing and signaling | MOLECULAR CELL | 2014 | Xin Cai |
| 13 | Activation of cyclic GMP-AMP synthase by self-DNA causes autoimmune diseases | PNAS | 2015 | Daxing Gao |
| 14 | Pan-viral specificity of IFN-induced genes reveals new roles for cGAS in innate immunity | NATURE | 2014 | John W Schoggins |
| 15 | Mitotic progression following DNA damage enables pattern recognition within micronuclei | NATURE | 2017 | Shane M Harding |

**S Table 2.** The top 10 occurrences and the top 10 new APY keywords in Figure 5.

| Rank | Keyword | Occurrences | Cluster | Keyword | APY | Cluster |
| --- | --- | --- | --- | --- | --- | --- |
| 1 | cGAS | 605 | 2 | ovarian cancer | 2020.82 | 1 |
| 2 | I IFN | 257 | 4 | neuroinflammation | 2020.64 | 3 |
| 3 | cGAS-STING pathway | 243 | 1 | nanoparticles | 2020.64 | 1 |
| 4 | STING | 227 | 1 | replication stress | 2020.60 | 1 |
| 5 | innate immunity | 152 | 2 | DNA repair | 2020.54 | 1 |
| 6 | DNA sensor | 140 | 2 | immunotherapy | 2020.47 | 1 |
| 7 | 2nd-messenger | 138 | 4 | chromosomal instability | 2020.43 | 1 |
| 8 | recognition | 134 | 2 | homologous recombination | 2020.36 | 1 |
| 9 | inflammation | 131 | 1 | therapy | 2020.33 | 1 |
| 10 | NF-κB | 122 | 2 | mitochondrial dysfunction | 2020.30 | 3 |
